# Precision Audiometry and Ecological Validity: Exploring the Link Between Patient-Reported Outcome Measures and Speech Testing in CI Users

**DOI:** 10.3390/audiolres15050142

**Published:** 2025-10-21

**Authors:** Matthias Hey, Thomas Hocke

**Affiliations:** 1 Audiology, ENT Clinic, UKSH, 24105 Kiel, Germany; 2 Cochlear Germany, 30539 Hannover, Germany; thocke@cochlear.com

**Keywords:** cochlear implant, speech intelligibility, signal processing, speech audiometry, ForwardFocus, temporal fluctuation, patient-reported outcome measure

## Abstract

Background/Objectives: Audiometric methods for hearing-impaired patients are constantly evolving as new therapeutic interventions and improved clinical standards are established. This study aimed to explore the relationship between patient-reported outcome measures in cochlear implant users and scores from audiometric test procedures in quiet and noise. Methods: In a prospective study, 20 postlingually deafened CI users were included. Speech comprehension was measured in quiet (by Freiburg words) and in noise (by the Oldenburg sentence test), while stationary speech-simulating or temporally fluctuating noise was applied and the noise sources were varied. Subjective feedback from the patients was obtained using the HISQUI19 questionnaire. Results: Word scores in quiet showed a significant positive correlation with the user’s subjective assessment of hearing ability using the questionnaire (Spearman’s R = 0.57). A greater correlation of the subjective evaluation of comprehension against fluctuating background noise as compared with stationary background noise was evident. On the other hand, the test–retest accuracy was reduced by a substantial factor in the transition from stationary to fluctuating background noise. Conclusions: By introducing temporal fluctuations in the background noise, the ecological validity can be improved, but at the cost of a parallel decrease in the accuracy of the test procedure. Especially in the context of studies, this knowledge may help to improve the choice of the specific test method used in evaluating the relationship between ecological validity and precision audiometry.

## 1. Introduction

Audiological methods such as speech-testing and patient-reported outcome measures (PROMs) are undergoing a perpetual evolution impelled both by new therapeutic interventions for hearing-impaired patients and by improved clinical standards. Consequently, these methods have to be evaluated and judged in the context of evolving therapeutic options, and therefore changing patient characteristics, corresponding to the intervention(s) used.

As early as the 1940s, the WHO emphasized that health encompasses physical, mental, and social well-being—not merely the absence of disease [1]. Patient-reported outcome measures (PROMs), which capture patients’ perspectives on their quality of life, emerged in the 1960s [2] and were later introduced into audiology [3]. Over recent decades, subjective assessments have gained importance [4,5], leading to the development of various tools such as questionnaires, visual analog scales, and ecological momentary assessments [6,7,8]. These instruments can be used to guide treatment for patients with conventional or implantable hearing systems, offering a cost-effective way to evaluate subjective outcomes. In this context, PROMs can reflect therapy-specific factors in the field of CI provision with respect to age and social support [5,9,10,11,12,13,14].

One pillar in German audiometry was developed by Hahlbrock [15,16]. This Freiburg monosyllabic test in quiet was designed for the clinical context of the time—primarily unilateral hearing aid fittings and surgery for conductive hearing loss. As therapeutic options expanded over the following decades, including bilateral hearing aids [17] and bone-anchored devices [18], audiometric testing evolved accordingly. Early studies already indicated that audiometric results and patient-reported outcomes may not always align [19,20].

With the introduction of cochlear (CI) and active middle-ear implants [21,22,23,24,25,26], the need for repeated and precise speech testing in quiet and noise became essential. This led to stricter requirements with respect to test–retest reliability, learning curves, ceiling effects, and clinical feasibility [27]. Simultaneously, the demand grew for tests that better reflect everyday listening, emphasizing ecological validity through speech-in-noise testing [6,28]. To address these challenges, Hagerman introduced the matrix sentence test [29], using grammatically correct but semantically meaningless sentences to reduce learning effects and improve reproducibility. Initially based on fixed-level testing, the method evolved with adaptive measurements [30], significantly reducing test time. The German version of this test [31,32] was later adapted for multiple languages [33]. Most impressively, these test procedures show a high precision in terms of test–retest reproducibility for measurement in stationary noise (DIN 8253 3) [34], e.g., for normal-hearing listeners, the reproducibility is 0.5 dB [33], while it is poorer for CI patients, being 1.1 dB [35]. In fluctuating noise, test–retest reproducibility further worsens by a factor of two to five [36].

As Pollack [28] noted, everyday competing signals are characterized by fluctuations in time, frequency, and space. Recent studies [37,38,39] explored how matrix test features could better reflect real-life hearing, deviating from standard protocols: for example, OlSa sentences were used with varied loudspeaker setups and with fluctuating noise, or even the test material itself, used as maskers. These approaches aimed to overcome the limitations of routine audiometry, particularly its weak correspondence with subjective evaluations.

To summarize, speech-in-noise testing is widely considered the gold standard in audiology for assessing real-world hearing performance. Its ecological validity is assumed to align closely with PROMs, which reflect subjective hearing experiences. However, previous research has found a correlation between the Freiburg monosyllabic word test in quiet (!) and PROMs of up to 0.7 [40]. This presents a surprising contradiction, as speech-in-noise tests are generally regarded as the more appropriate method for capturing real-life hearing challenges [6] compared to monosyllables in quiet [41].

Consequently, this study was designed to evaluate the relationship between PROMs and speech audiometry in CI users in the presence of this contradiction: Do less complex and less ecologically valid considered tests capture the aspects of hearing that matter most to patients? This represents a certain tension between precision audiometry, which prioritizes reproducibility and control, and ecological validity, which aims to reflect everyday listening conditions.

The study is the third in a series involving the same patient cohort, with earlier publications focusing on speech comprehension [8,42], but with this one focusing on ecological validity. Different noise types and spatial speaker configurations were applied for the use of Oldenburg sentences in addition to the Freiburg monosyllables in quiet, both representing the most common used speech tests in Germany [43]. The corresponding PROM was assessed using the HISQUI19 [44,45].

## 2. Materials and Methods

### 2.1. Study Design

This study was performed according to a single-subject design with repeated measurements. Feedback was obtained from patients by using the German version of the Hearing Implant Sound Quality Index (HISQUI19) questionnaire [44,45]. The HISQUI-19 is a freely available (currently in at least eight languages) validated questionnaire designed to measure how cochlear implant users perceive their hearing improvement in everyday situations. Consisting of 19 items rated on a 7-point Likert scale, it is unaffected by age, gender, implant type, or duration of hearing loss. It is validated and reliable tool to assess CI users’ self-perceived sound quality. Due to its compressed form, patient’s feedback may be captured easy within 10 min. Its standardized format allows for comparisons across studies and populations, while it covers a wide range of real-life listening situations.

The in-lab characterization of speech communication abilities was performed using a set of speech tests in quiet (monosyllabic words) as well in noise (adaptive matrix test). To reconstruct everyday listening situations for the clinic in a systematic way, the temporal characteristics of the noise and the spatial arrangement of the signal sources were varied.

### 2.2. Research Participants

Twenty participants were recruited. The investigation was approved by the local University Ethics Committee (study number D 6/18). Written informed consent was obtained from all subjects involved in the study. All procedures were performed in accordance with the ethical standards of the institutional and national research committees and with the 1964 Helsinki declaration and its later amendments or comparable ethical standards.

All patients were adults with post-lingual onset of deafness. Each had received a cochlear implant model Nucleus CI24RE or from the CI5 series (Cochlear Limited, Australia). All were experienced CI users and had utilized a CP800 series sound processor for more than five years. To be included in this study, participants were required to have scores of 80% or more in the Oldenburg sentence test in quiet at 65 dB_SPL_ [35]. At the time of the examination, the patients had an average age of 53 years (range: 31–76 years) and an average experience with CI of 8 years (range: 5–15 years). Ten of them were female and ten were male. A detailed description of the participants can be found in [42].

### 2.3. Test Procedures

After starting with the familiar CP800 device, the following speech processor generations were used and tested in the different settings in random order. After examination with the CP800 processor, patients were alternately fitted with either the CP900 or CP1000 processor according to a predefined randomization table so that the transition began initially with equal proportions of each processor. Patients used each of the newer speech processors during a take-home period of two to three weeks for accustomization before testing in the clinic. For each speech processor generation, the patients completed the HISQUI19. The speech scores of the different speech processors have been published elsewhere [42] and are not the focus of this contribution.

All tests were conducted in an audiometric test booth [46]; sound was introduced through calibrated loudspeakers placed 1.3 m away from the patient. Bilateral CI users were tested on one ear while the contralateral sound processor was switched off.

All speech comprehension tests were presented using a computer-based audiometer (Equinox; Interacoustics, Denmark; with evidENT 3 software, Merz Medizintechnik, Germany). Two speech test were used according to the German CI guideline [47] and a survey which identified them as most commonly used test in tertiary centers [43]. For speech in quiet, the Freiburg monosyllabic words were applied frontally [15] at presentation levels of 50 and 65 dB_SPL_ for each speech processor. Items were presented in randomized order to minimize any repetitive learning effect.

For speech comprehension in noise, the German version of a matrix test was used: the Oldenburg sentences [48]. Each list contained 30 sentences. It was measured using an adaptive procedure [32], with the aim of determining the SRT (i.e., the signal-to-noise ratio, SNR, yielding a score of 50% words correct). All CI recipients were accustomed to the test procedure, having previously been assessed using this test several times as part of our clinical routine. To reduce the procedural learning effect with the Oldenburg sentence test [35], a training test was performed (one list of 30 sentences) before each session. According to Hey et al. [35], recipients were only included if they showed an SRT in S0N0 (stationary noise) better than 3dBSNR.

Speech comprehension in noise was tested while the signal-source location was varied: (A) speech and noise from the front, S0N0; (B) speech from the front and ipsilateral noise, i.e., coming from the side of the CI, S0NCI; and (C) speech from the front and noise presented non-coherently from three loudspeakers placed in the rear hemisphere at 90°, 180°, and 270° (S0N3). Additionally, the temporal characteristics of the competing signal source were changed: the speech-simulating stationary noise of the Oldenburg sentence test [48] was replaced by a fluctuating speech-simulating noise with the spectral properties of a male speaker [49], the ICRA5 noise.

Changing the various speech processors was associated with using different pre-processing algorithms while leaving the individual map parameters stable (e.g., T- and C-levels).

To yield individual subjective feedback on sound quality while the CI sound-processors were in use, the HISQUI19 [44] was used on the same day as the speech testing. Patients were required to answer each of the 19 questions on a 7-step scale. Quantification was performed by summing the results of all 19 questions, yielding a score ranging from 19 to 133.

Data are presented as scatter plots. For correlation analyses, Spearman’s rank analysis was used. The correlation coefficients were compared using Fisher’s z transformation. The Bonferroni–Holm method was used to correct for multiple testing.

## 3. Results

All patients were able to perform the speech tests in quiet and noise. The questionnaire was completed by each patient on the day of audiometric measurements in the clinic.

In the study population, the mean ‘word correct’ score at 65 dB_SPL_ in quiet was (86 ± 15)% and SRT for the Oldenburg sentences (S0N0 stationary noise) was (–2.1 ± 1.7) dB_SNR_. Subjective rating by patients using the HISQUI19 ranged from 48 to 123, with the mean and median both 91.

**Figure 1:**
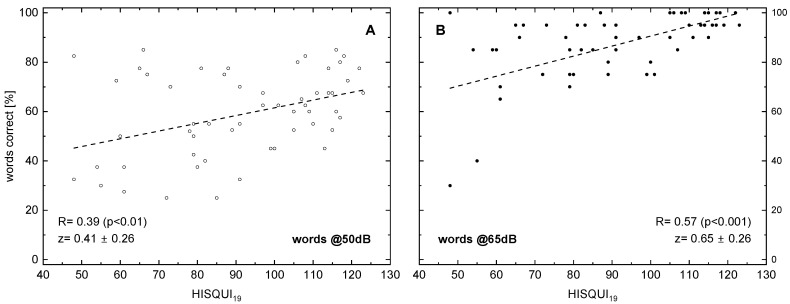
Word recognition score (monosyllabic Freiburg words) in relation to the hearing implant sound quality index, HISQUI19, for presentation levels of 50 dB_SPL_ (**A**) and 65 dB_SPL_ (**B**). Dotted lines indicate the results of linear regression; the corresponding correlation coefficients are shown. In addition to the Spearman correlation coefficients R, the z transforms according to Fisher and their 95%confidence intervals are shown. The *p*-values were corrected for multiple testing.

The following figures display the results of different speech audiometry test conditions to the results of the HISQUI19. When testing speech comprehension in quiet, we see, in Figure 1, for 50 and for 65 dB, a significant correlation with the HISQUI19 score, with R = 0.39 and 0.57, respectively.

**Figure 2:**
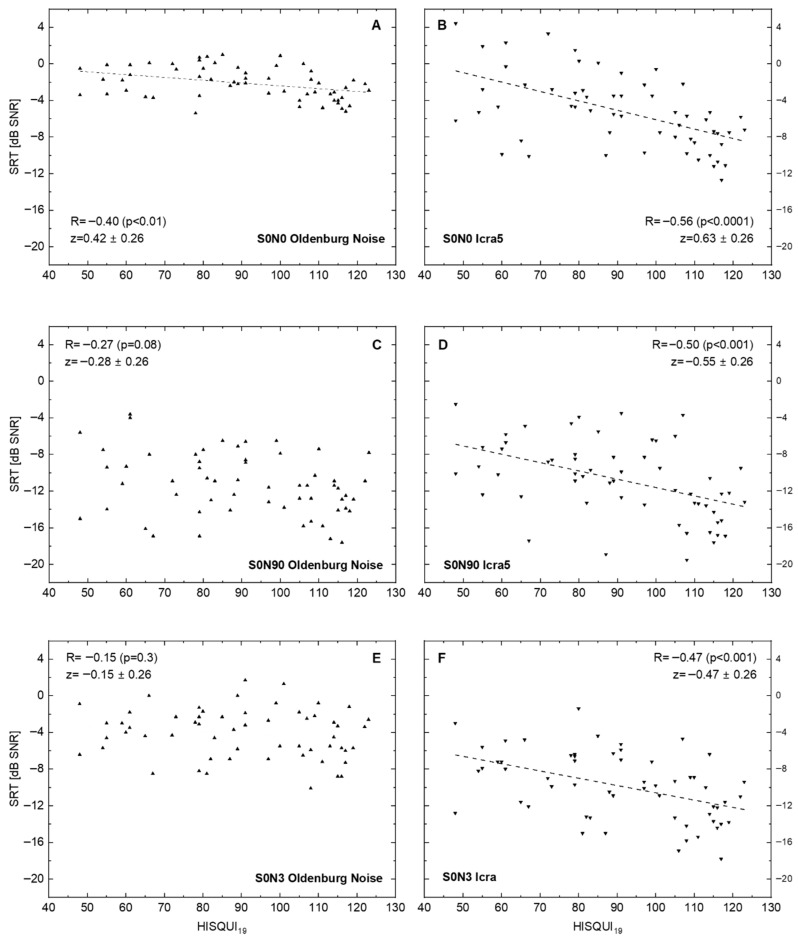
Speech recognition threshold (SRT for Oldenburg sentences) in relation to the hearing implant sound quality index, HISQUI19, for speech-simulating stationary Oldenburg noise (**A**,**C**,**E**) and for the fluctuating noise ICRA5 (**B**,**D**,**F**). Dotted lines show linear regression for significant correlations. (**A**,**B**): Signal and noise are presented from the front. (**C**,**D**): Signal is presented from the front and noise ipsilaterally to the CI. (**E**,**F**): Signal is presented from the front and non-coherent noise from three loudspeakers in the rear hemisphere: 90°, 180°, and 270° ipsilaterally to the CI. In addition to the Spearman correlation coefficients R, the z transforms according to Fisher and their 95%confidence intervals are shown. The *p*-values were corrected for multiple testing.

Figure 2 shows the results of ‘speech comprehension in noise’ tests with three different spatial loudspeaker settings. Additionally, for the disturbing noise source, the temporal characteristic was changed from the established stationary speech simulating (the Oldenburg sentence test noise) to a noise fluctuating in time. This resulted in six data sets. Correlation of SRT in noise with the patients’ own HISQUI19 ratings increased for all spatial settings from stationary to fluctuating noise. The best correlation between the two measures was obtained for the condition S0N0.

The correlation between speech comprehension and the patients’ subjective rating decreased with increased spatialness (wider placing of the loudspeakers). At the end, for the situation S0N3, in stationary noise, no significant correlation between the two parameters measured was seen (R = –0.15; *p* = 0.3), whereas there was a significant negative correlation (R = –0.47; *p* < 0.001) seen for fluctuating noise in this spatial setting.

## 4. Discussion

In our investigation, we compared speech recognition in quiet and noise in a population of CI users under different audiometric conditions with a chosen patient-reported outcome measure, the HISQUI19. The analysis of PROMs and audiometric results did not completely yield the expected correlations across the different audiometric conditions, as we used the following spatial settings, a masker, and the speech material:

- The change from stationary to fluctuating noise resulted in a higher correlation of SRTs and PROMs.
- If stationary or fluctuating noise was used, an increased spatialness did not result in a higher correlation of SRTs and PROMs.
- Monosyllables in quiet showed a correlation of R = 0.57, which was the highest of the tested conditions.

This is remarkable, as monosyllabic word tests in quiet inherit known limitations in test–retest reproducibility and—as known for the German test—for phonemic as well as perceptual balance [50,51].

Our study is in contradiction to the widely established assumption that sentence tests are much more representative than monosyllable tests for realistically simulating communication situations [52]. The high correlation of the monosyllabic score in Figure 1 does not support this hypothesis.

Although spatial listening conditions are often preferred in studies to demonstrate the benefits of signal processing technologies such as multi-microphone systems, our data did not show a corresponding change in patient-reported outcomes, even though a number of studies show a considerable benefit in speech scores, respectively, SRTs. The reasons for this remain unclear based on the current data set. It is conceivable that patients tend to avoid situations where signal processing is most active. Additionally, the temporal gap between the experienced benefit and the assessment of PROMs introduces a bias. These limitations can be avoided in future studies using ecological momentary assessment in corresponding listening environments.

With regard to the requirements of speech tests in general [33], the matrix test in stationary noise fulfills clinical requirements such as a high slope value, small variability across test lists, and small test–retest differences, resulting in good test–retest reproducibility [34]. These obvious advantages are diminished for fluctuating noise, showing more than halved slope values and more than doubled test–retest differences [36].

To summarize, our results strongly indicate a trade-off between ecological validity (as described by the patient’s PROM) and precision audiometry (described by good test–retest reproducibility). When less precise audiometric methods are used—such as measuring SRT in fluctuating noise and monosyllabic Freiburg words in quiet—the speech audiometric test results show a closer correspondence with everyday life aspects of patients. The other approach is precision audiometry, which is best realized by Oldenburg sentences in stationary noise for the S0N0 situation.

It is therefore very important to choose the audiometric setup according to the scientific or therapeutic question, so it might be advisable to choose an audiometric procedure with a higher degree of ecological validity if the outcome in a given patient population is investigated at group level. In contrast, when, e.g., focusing on the quality of a noise-canceling algorithm with impact in the individuals’ speech recognition, it might be a good choice to select an audiometric procedure with the best available test–retest accuracy.

To summarize the methodological dilemma: all approaches to improve the ecological validity according to Keidser et al. [6] are in contrast to the design assumptions for precision audiometry [33]. High ecological validity is characterized by, e.g., transient measurement conditions such as changing intonation, spectral and temporal fluctuations, different speakers, and reverberation, while precision audiometry relies on, e.g., optimized construction of the masking signal, high slope values, and homogeneous intelligibility. All of the above-mentioned characteristics of precision audiometry result in maximum stationarity, which is in contrast to the demands of ecological validity.

### Limits of the Study and Possible Improvements

A disadvantage of the Oldenburg sentence test is the fact that not all patients are able to perform this procedure because of the high rate of speaking or because the test may not be performed in their native language. It is known that about 80% of the CI recipients in an actual clinical patient group are able to cope with adaptive SRT measurements in noise [53]. A questionnaire is much simpler to realize. Nearly all people are able to fill out a PROM questionnaire of this type.

Both the tools investigated in this study (audiometry and questionnaire) have the inherent disadvantage of—or at least are biased by—transferring a supposed everyday aspect into a clinical setting. A continuing alternative to questionnaires would be the use of EMA tools. New technologies like tablets and smartphones may potentially offer a promising platform to allow for momentary feedback from a patient in a given relevant situation [54]. To our knowledge, there are no established EMA tools that are broadly usable in audiology. Such tools would represent a further meaningful step beyond the application of questionnaires, even with all of their known disadvantages such as recall bias [6]. They would reveal an immediate patient response in situ.

The HISQUI19 includes multiple dimensions of hearing experience, but the established analysis uses only the total score. This avoids type I errors; however, it may potentially mask important patterns in the subscales. An item-wise analysis could be subject to future studies.

The number of subjects limits statistical power and generalizability. Finally, all participants were experienced CI users with high baseline performance (≥80% in quiet). This may have introduced a ceiling effect, and potentially limits applicability to broader CI populations, especially new or poorer-performing users.

## 5. Conclusions

Among the audiometric procedures assessed in quiet and noise, the monosyllabic test in quiet emerged as a still relevant measure, showing a correlation of 0.57 with patient-reported outcomes. These findings question the common assumption that sentence tests in noise inherently offer greater ecological validity and therefore naturally align better with subjective benefit.

Regarding speech-in-noise testing, our results highlight a trade-off between measurement precision (e.g., test–retest reliability) and ecological validity (e.g., alignment with PROMs).

In accordance with previous studies, fluctuating noise shows a higher correlation with PROMs than stationary noise, but inherits a poorer test–retest-reliability. Depending on the clinical or research objective, either aspect can be prioritized. Consequently, the choice of audiometric method should be guided by the specific therapeutic or investigative context.

## Data Availability

The original contributions presented in this study are included in the article. Further inquiries can be directed to the corresponding author(s).
